# Serum and hair zinc levels in breast cancer: a meta-analysis

**DOI:** 10.1038/srep12249

**Published:** 2015-07-16

**Authors:** Xiujuan Wu, Jing Tang, Mingjun Xie

**Affiliations:** 1 The First People’s Hospital of Yibin, Yibin, Sichuan Province, 644000, China; 2 Luzhou Medical College, Luzhou, Sichuan Province, 646000, China

## Abstract

Many studies have investigated the association between serum/hair zinc levels and breast cancer, but the results were inconsistent. To compare the serum and hair zinc levels in women with breast cancer and controls, we conducted a systematic literature search of PubMed, Web of Science, Cochrane Library and Embase to identify relevant studies with publication dates up through November 2014. Based on a random effects model, summary standard mean differences (SMDs) and the corresponding 95% confidence intervals (CIs) were used to compare the serum and hair zinc levels in women with breast cancer and controls. Fourteen studies that investigated serum zinc levels and seven studies that assessed hair zinc levels were included. Our study observed no difference in serum zinc levels between breast cancer cases and controls (SMD (95%CI): −0.65[−1.42,0.13]). However, we determined that hair zinc levels were lower in women with breast cancer compared with those of controls (SMD (95%CI): −1.99[−3.46, −0.52]). In conclusion, this study was the first to provide evidence that hair zinc levels in female breast cancer patients are lower than in controls; however, there was no significant difference in serum zinc levels between female breast cancer patients and controls.

Breast cancer is a common disease and the second leading cause of death among women in Canada and America^1^. Moreover, breast cancer incidence has significantly increased over the past two decades in Chinese females^2^. Although many studies have focused on breast cancer aetiology, nosogenesis and treatment, various aspects of this disease remain unknown. However, it is believed that trace elements play important roles in biological processes relevant to breast cancer, especially those elements that are essential components of antioxidants^3^,^4^. As an activator of many enzymes involved in the synthesis of DNA and RNA, zinc has been the subject of investigations regarding its importance in biochemical processes and antioxidant defence. Some studies have shown that zinc can induce apoptosis in cancer cells and inhibit cell proliferation^5^. High levels of zinc supplementation had a positive effect on reducing oxidative stress and improving immune responses in cancer patients^7^. However, some studies have indicated that zinc serves as a co-factor for cancer cell fission and replication^8^.

Kuo *et al.* showed that the serum zinc levels in women with breast cancer were lower than those in healthy controls^9^, and a similar result was reported for hair zinc levels^10^. However, in 1991, another study showed higher plasma zinc levels in healthy people were related to a higher subsequent breast cancer risk^11^. Furthermore, a Chinese population-based case-control study reported significantly higher hair zinc levels in breast cancer patients compared with those of controls^8^. However, another study determined that both serum and hair zinc levels were not different in women with breast cancer when compared with those in women without breast cancer^12^. Based on the results of these studies, whether there were differences in serum and hair zinc levels between women with breast cancer and controls was still unclear. Hence, we conducted a meta-analysis to clarify these conflicting results and compare the serum and hair zinc levels in women with breast cancer and controls.

## Methods

### Literature search

The literature searches were conducted using articles with publication dates through November 2014 from the PubMed, Web of Science, Cochrane Library and Embase databases with the following search terms: “zinc”, “trace elements”, “zinc levels” in combination with “breast cancer”, and “breast carcinoma”. The references of the relevant studies were searched for additional potential studies. If necessary, we contacted the authors of the original studies for the required data.

### Study selection

The studies that met the following criteria were included: 1, the study assessed serum and hair zinc levels in breast cancer patients and controls; 2, all cases of breast cancer were diagnosed by pathological biopsy or other standard methods, and the controls were females without breast cancer; and 3, biological samples (serum and hair) were obtained before therapeutic interventions. Initially, we reviewed relevant titles and abstracts to ascertain a potential fit for the inclusion criteria. In the presence of uncertainty regarding the relevancy, the full text of the article in question was examined. Because the data included in our study were taken from the literature, approval from an ethics committee was not needed.

### Data extraction and quality assessment

We used a standardized data extraction form to collect the data. The following information was collected from each of the included studies: the last name of the first author, publication year, study population, mean age of the participants, number of cases and controls and zinc concentrations.

The Newcastle-Ottawa Scale (NOS) was used to assess the quality of the studies^13^. The NOS ranges from 0 to 9 stars. Studies with less than 4 stars were excluded, whereas those with more than 6 stars were considered to be high quality studies.

Two independent authors conducted all of the above procedures, and any disagreements were resolved by discussion.

### Statistical analysis

The standard mean differences (SMDs) and the corresponding 95% confidence intervals (CIs) were used to compare the serum and hair zinc levels in women with breast cancer and controls. Homogeneity testing was performed with the Q and I^2^ statistics. Additionally, we conducted subgroup analysis to identify the association between the serum and hair zinc levels and other relevant study characteristics, such as geographical location. Sensitivity analysis was used to investigate the influence of a single study on the overall effect estimate by omitting one study at a time during repeated analyses. The Begg and Egger’s test were used to detect the evidence of a publication bias. In our study, P-values less than 0.05 were considered statistically significant. All analyses were conducted using STATA version 12.0.

## Results

### Literature search and study characteristics

**Figure 1:**
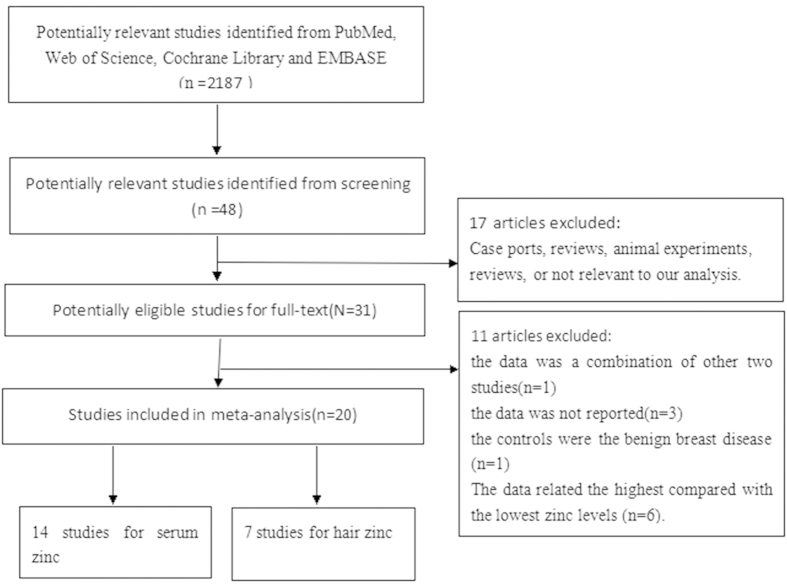
Flow chart of study selection

We initially retrieved unique citations from PubMed, Web of Science, Cochrane Library and Embase. After screening the abstracts or titles, most studies were excluded mainly because they were reports, animal experiments, or reviews or were not relevant to our analysis. After searching, we included 14 studies^9^,^12^,^14^,^15^,^16^,^17^,^18^,^19^,^20^,^21^,^22^,^23^,^24^,^25^ (including 662 cases and 775 controls) that assessed serum zinc levels and 7 studies^8^,^10^,^12^,^26^,^27^,^28^,^29^ (including 264 cases and 449 controls) that assessed hair zinc levels. Figure 1 presents the flow chart of study selection.

All studies had NOS scores greater than 6. Most of these studies were conducted among Asian and European females except two studies that were not^22^,^23^. All studies reported zinc levels as the mean ± sd. Table 1 presents the general data from the included studies.

### Serum zinc levels and breast cancer

**Figure 2:**
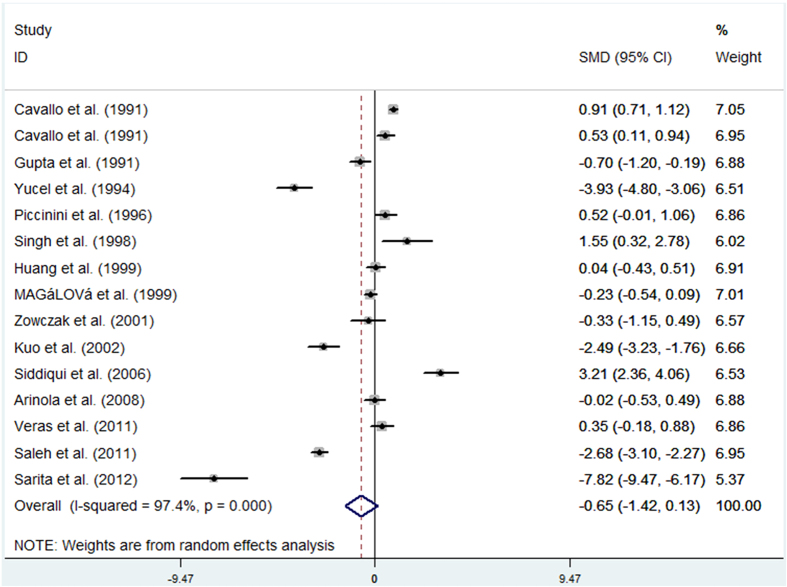
Forest plot showing the meta-analysis outcome of the serum zinc levels for breast cancer patients versus healthy controls.

Data from 14 studies were analysed in a random-effects model to compare the serum zinc levels in women with breast cancer and controls. The results suggested that there was no significant difference in serum zinc levels between patients with breast cancer and controls (SMD (95%CI): −0.65[−1.42,0.13]). Significant heterogeneity was found among these studies (P < 0.05, I^2^ = 97.4%). Figure 2 presents the results of this analysis.

Subgroup analysis indicated that there was no difference in serum zinc levels between the breast cancer cases and controls among Asians, Europeans and other populations. The results of this subgroup analysis are presented in Table 2.

**Figure 3:**
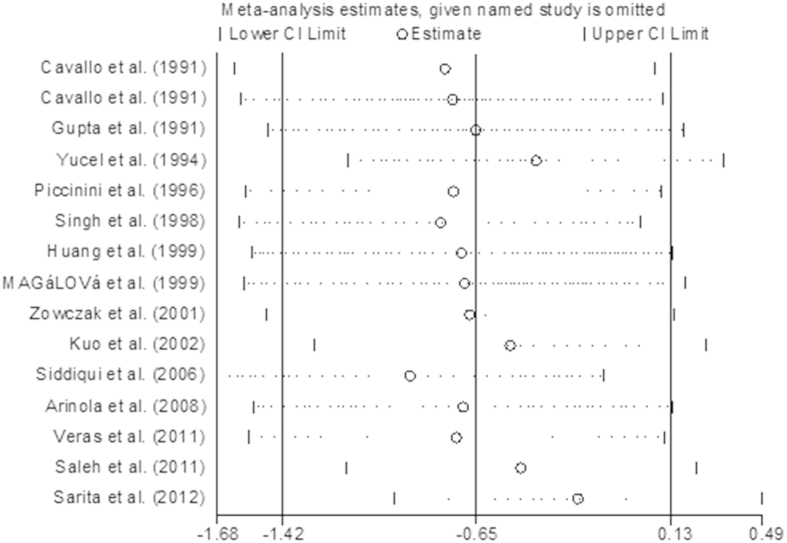
Sensitivity analysis for studies in serum zinc for breast cancer patients versus healthy controls.

Sensitivity analysis showed that when the study conducted by Siddiqui *et al.* was omitted, the combined SMD changed, this meant that this study impacted the combined result significantly. The results of the sensitivity analysis are presented in Fig. 3.

**Figure 4:**
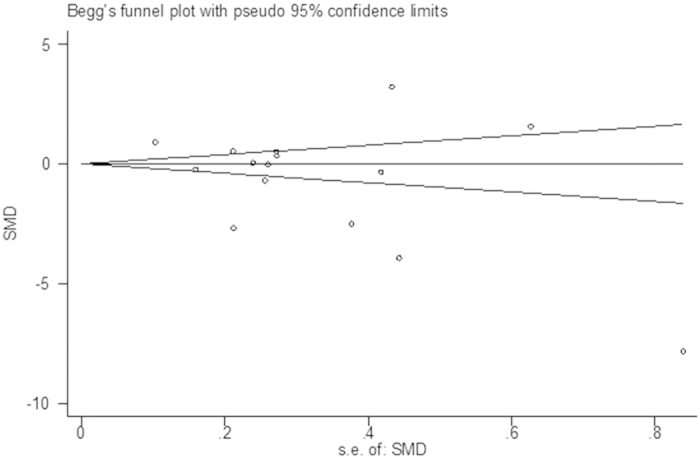
Publication bias for studies in serum zinc for breast cancer patients versus healthy controls.

According to the shape of the funnel plots and the results of the Begg and Egger’s test, we found no evidence of publication bias (p = 0.110) (Fig. 4).

### Hair zinc levels and breast cancer

**Figure 5:**
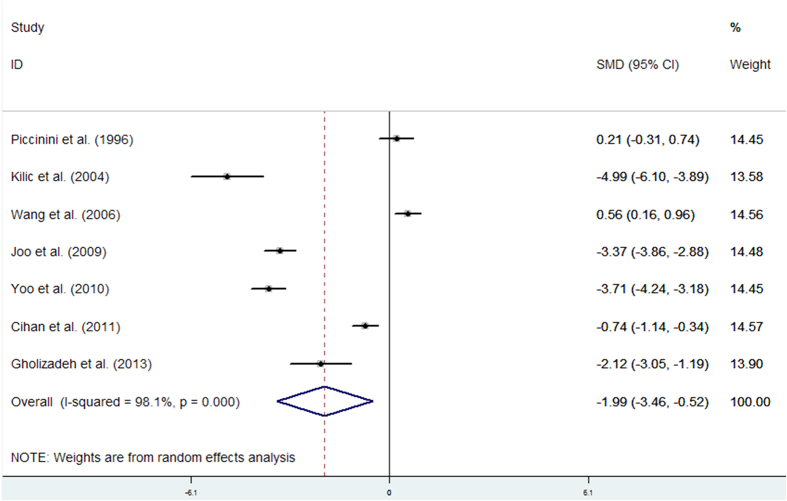
Forest plot showing the meta-analysis outcome of the hair zinc levels for breast cancer patients versus healthy controls.

Seven studies were analysed in a random-effects model to compare the hair zinc levels in women with breast cancer and controls. We found that hair zinc levels in breast cancer cases were significantly lower than in the controls (SMD (95%CI): −1.99[−3.46, −0.52]). We also observed strong evidence of heterogeneity (I^2^ = 98.1%, P < 0.05). These results are presented in Fig. 5.

Subgroup analysis indicated that the lower levels of hair zinc in the breast cancer cases were also observed among Asian women (SMD (95%CI): −2.37[−4.01, −0.72]). A similar result was not observed in studies of European women; however, only one study of European women was included in the analysis (Table 2).

**Figure 6:**
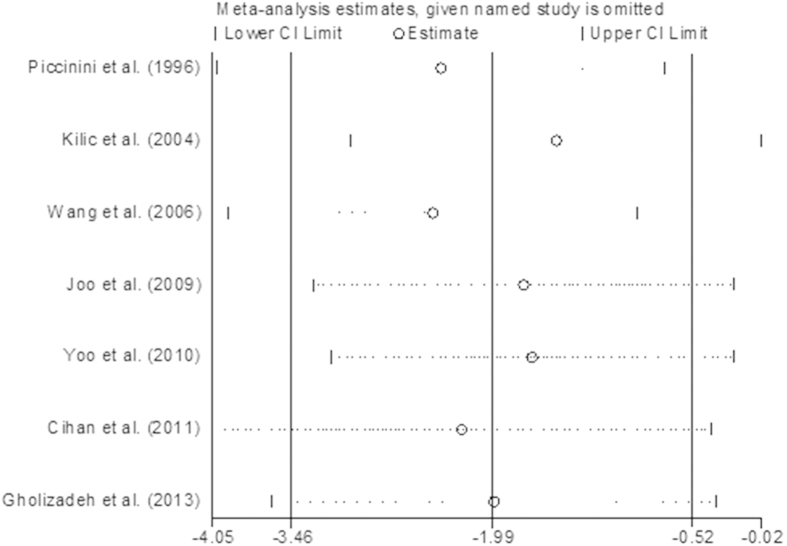
Sensitivity analysis for studies in hair zinc for breast cancer patients versus healthy controls.

**Figure 7:**
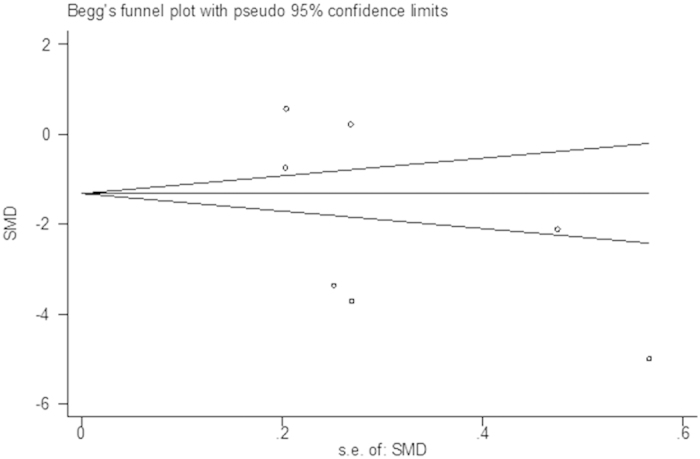
Publication bias for studies in hair zinc for breast cancer patients versus healthy controls.

The sensitivity analysis showed that the combined SMDs were all statistically significant and similar to one another, and no one study significantly influenced the combined SMD (Fig. 6). The Begg’s and Egger’s regression test showed no evidence of publication bias (p = 0.232) (Fig. 7).

## Discussion

Our study illustrated that there was no difference in serum zinc levels between breast cancer patients and controls; however, we found evidence of lower hair zinc levels in breast cancer patients when compared with those of controls.

Zinc is important for the function of numerous cellular processes and critical for growth; however, it may also play an important role in cancer aetiology and outcome^30^. The relationship between zinc and cancer aetiology and progression has been extensively studied with contradictory results. One review reported that zinc supplementation had beneficial effects on cancer by decreasing angiogenesis and induction of inflammatory cytokines while increasing apoptosis in cancer cells^31^. However, other studies have proposed that zinc is essential to rapidly growing tissues and seems to act protectively on the growth of both normal and cancer cells^32^.

Hair may serve as a good biopsy material for assessment of trace elements status^33^. In other types of tumours, hair zinc levels were significantly lower in tumour patients than in controls^34^,^35^. In 2000 and 2002, two studies proposed that hair might be a promising tool for the diagnosis of breast cancer patients^36^,^37^. Zinc serves an important role in breast cancer during the cancer cell fission and replication, and the polymerase needs zinc to serve as a co-factor for activation. Therefore, as breast cancer cells rapidly increase, they consume large amounts of zinc resulting in lower zinc concentrations in hair^8^. Hence, the hair zinc levels in patients with breast cancer are lower than in controls. Our study also suggested that there was a lower hair zinc level in the breast cancer patients, which supports the hypothesis that breast cancer cells consume more zinc, thus reducing hair zinc levels.

However, we could not identify any differences in serum zinc levels between breast cancer patients and controls. The explanation for this result is unknown, but may be that serum biochemistry is dynamic and serum concentrations of zinc are susceptible to numerous physiopathological effects in response to various internal stimuli such as stress, infection, hormones and diet^38^,^39^,^40^,^41^. This makes serum zinc a less ideal source of biomarkers in humans. Hence, some studies considered the analysis of zinc levels in the erythrocyte compartment to be more precise than that in the serum^42^.

To the best of our knowledge, this report describes the first comprehensive study to estimate the difference in hair zinc levels between breast cancer patients and controls. However, several limitations of our study should be considered. First, these results may reflect some potential contamination of the hair from environmental factors and shampoos, which may contain pyrithione zinc. These factors may influence the hair zinc levels in patients and controls, however, no included studies adjusted for this confounding factor. Second, the number of cases and controls in each study was relatively small, and several studies were excluded due to a lack of control data. Although our study combined many studies, the sample size was still small. Third, information from other variables such as nutrition, lifestyle and family history was lacking. Fourth, strong evidence of heterogeneity among the studies was observed. The following reasons may partially explain this heterogeneity: 1, inconsistent methods of zinc measurement; 2, inconsistent standard units for measuring zinc levels; 3, other variables such as hormone receptor expression, menopausal status, and family history were not fully considered; 4, the subjects were from different countries and they had inconsistent diets and lifestyles; and 5, although there was little evidence of publication bias among these studies, some negative studies which were not published should be taken into consideration. Finally, most of the included studies were conducted in Asian and European women, and whether the results of our study would apply to other populations is unclear.

## Conclusions

The present study provides evidence that there is a significant difference in hair zinc levels between individuals with and without breast cancer. However, we did not find any difference in serum zinc levels between breast cancer patients and controls.

## Additional Information

**How to cite this article**: Wu, X. *et al.* Serum and hair zinc levels in breast cancer: a meta-analysis. *Sci. Rep.* **5**, 12249; doi: 10.1038/srep12249 (2015).

## Figures and Tables

**Table 1:** The characteristics of included studies.

Study(Year)		**Breast cancer patients**			**Healthy controls**		
	**Areas**	**Mean age**	**N**	**Zn(mean ± SD)**	**Mean age**	**N**	**Zn(mean ± SD)**
Serum zinc levels							
Cavallo *et al.*(1991)	France	53.10	47	90.8 ± 10.3	53.10	46	85.4 ± 10.2
	Italy	49.60	206	101.8 ± 15.8	47.10	212	87.9 ± 14.6
Gupta *et al.*(1991)	India	47.00	35	99.4 ± 26.5	46.00	30	115.1 ± 16.1
Yucel *et al.*(1994)	Turkey	48.00	31	0.479 ± 0.130	50.00	30	1.355 ± 0.289
Piccinini *et al.*(1996)	Italy	58.60	38	86.43 ± 23.46	56.80	22	74.03 ± 24.22
Singh *et al.*(1998)	India	N	10	26.3 ± 5.3	N	5	17.0 ± 7.3
Huang *et al.*(1999)	China	48.00	35	1.14 ± 0.28	44.50	35	1.13 ± 0.17
Magálová *et al.*(1999)	Slovakia	55.6	73	14.54 ± 2.11	51.7	87	14.99 ± 1.89
Zowczak *et al.*(2001)	Poland	N	8	13.0 ± 3.3	N	21	13.8 ± 2.0
Kuo *et al.*(2002)	China	N	25	753.77 ± 86.35	N	26	976.734 ± 92.25
Siddiqui *et al.*(2006)	India	46.84	25	8.49 ± 1.53	42.37	25	4.74 ± 0.62
Arinola *et al.*(2008)	Nigeria	47.17	29	143.27 ± 6.62	46.07	30	143.38 ± 7.54
Veras *et al.*(2011)	Brazil	N	29	69.69 ± 9.0	N	26	65.93 ± 12.44
Saleh *et al.*(2011)	Kuwait	47.20	50	0.99 ± 0.39	46.90	150	3.6 ± 1.1
Sarita *et al.*(2012)	India	N	21	13.0 ± 2.0	N	30	38.5 ± 3.9
Hair zinc levels							
Piccinini *et al.*(1996)	Italy	58.60	38	162.30 ± 27.20	56.80	22	156.32 ± 30.07
Kilic *et al.*(2004)	Turkey	53.00	26	147.0 ± 21.40	55.00	27	293 ± 35.15
Wang *et al.*(2006)	China	54.20	50	247.6 ± 121.7	52.20	50	195.9 ± 50.2
Joo *et al.*(2009)	Korea	47.10	40	145 ± 7.23	47.80	144	163.54 ± 4.93
Yoo, *et al.*(2010)	Korea	47.08	37	14.33 ± 0.72	47.75	144	16.35 ± 0.49
Cihan *et al.*(2011)	Turkey	50.30	52	29.374 ± 20.367	47.40	52	63.7 ± 6.187
Gholizadeh *et al.*(2013)	Iran	N	21	12.3 ± 5.3	N	10	24.0 ± 6.0

**Table 2:** The results of subgroup analyses.

**Subgroup**	**NO. Of date from studies**	**SMD(95%CI)**	**I^2^**	**P value for heterogeneity**
Serum Zn geographical location				
Asian countries	8	−1.54[−3.13,0.04]	97.8%	0.000
European countries	4	0.32[−0.23,0.86]	90.2%	0.011
Other countries	2	0.16[−0.21,0.53]	0.0%	0.333
Hair Zn geographical location				
Asian countries	6	−2.37[−4.01,−0.72]	98.2%	0.000
European countries	1	0.21[−0.31,0.74]	–	
